# Linoleic Acid Promotes Mitochondrial Biogenesis and Alleviates Acute Lung Injury

**DOI:** 10.1111/crj.70004

**Published:** 2024-09-23

**Authors:** Jie Liu, Yu Jiang, Qiuhong Zhang, Yin Qin, Kexin Li, Yu Xie, Tingting Zhang, Xiaoliang Wang, Xi Yang, Li Zhang, Gang Liu

**Affiliations:** ^1^ Department of Emergency and Critical Care Medicine University‐Town Hospital of Chongqing Medical University Chongqing China; ^2^ Medical Sciences Research Center University‐Town Hospital of Chongqing Medical University Chongqing China; ^3^ Department of Respiratory and Critical Care Medicine University‐Town Hospital of Chongqing Medical University Chongqing China; ^4^ Department of Anesthesiology Chongqing Emergency Medical Center Chongqing China; ^5^ Department of Pathophysiology Chongqing Medical University Chongqing China

**Keywords:** acute lung injury, inflammation, linoleic acid, lipopolysaccharide, mitochondrial biogenesis, PGC‐1α

## Abstract

**Introduction:**

Acute lung injury (ALI) is a critical and lethal medical condition. This syndrome is characterized by an imbalance in the body's oxidation stress and inflammation. Linoleic acid (LA), a polyunsaturated fatty acid, has been extensively studied for its potential health benefits, including anti‐inflammatory and antioxidant activities. However, the therapeutic effects of LA on ALI remain unexplored.

**Methods:**

Lipopolysaccharide (LPS), found in gram‐negative bacteria's outer membrane, was intraperitoneally injected to induce ALI in mice. In vitro model was established by LPS stimulation of mouse lung epithelial 12 (MLE‐12) cells.

**Results:**

LA treatment demonstrated a significant amelioration in LPS‐induced hypothermia, poor state, and pulmonary injury in mice. LA treatment resulted in a reduction in the concentration of bronchoalveolar lavage fluid (BALF) protein and an increase in myeloperoxidase (MPO) activity in LPS‐induced mice. LA treatment reduced the generation of white blood cells. LA treatment reduced cell‐free (cfDNA) release and promote adenosine triphosphate (ATP) production. LA increased the levels of superoxide dismutase (SOD) and glutathione (GSH) but decreased the production of malondialdehyde (MDA). LA treatment enhanced mitochondrial membrane potential. LA attenuated LPS‐induced elevations of inflammatory cytokines in both mice and cells. Additionally, LA exerted its protective effect against LPS‐induced damage through activation of the peroxisome proliferator‐activated receptor γ coactivator l alpha (PGC‐1α)/nuclear respiratory factor 1 (NRF1)/transcription factor A of the mitochondrion (TFAM) pathway.

**Conclusion:**

LA may reduce inflammation and stimulate mitochondrial biogenesis in ALI mice and MLE‐12 cells.

## Graphical abstract

According to our results, LA treatment demonstrated a protective effect on LPS‐induced ALI in both in vivo and in vitro experiments. LA exerted its protective effects is by enhancing the PGC‐1α/NRF1/TFAM signaling pathway. This pathway plays a critical role in mitochondrial biosynthesis. Overall, our findings suggest that LA treatment holds promise as a potential therapeutic strategy for managing LPS‐induced ALI.

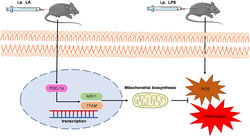

## 1 Introduction

Acute lung injury (ALI) is a severe and often fatal condition that typically occurs as a result of infection [1]. It is characterized by augmented vascular permeability, pulmonary edema, impaired gas exchange, and ultimately respiratory fail [2]. The pathogenesis of ALI involves dysregulated inflammation, where there is an abnormal immune response that leads to an overproduction of proinflammatory factors [3]. These inflammatory molecules further contribute to tissue damage and disruption in the lungs [4]. Excessive oxidative stress also significantly worsens the observed pathophysiological changes in ALI. In ALI, elevated levels of reactive oxygen species (ROS) occur due to oxidative stress, which can cause cellular damage and trigger inflammatory responses [5]. The sources of ROS generation include activated immune cells such as neutrophils and macrophages, as well as mitochondrial dysfunction. Given these insights into the pathogenesis of ALI, it becomes evident that targeting uncontrolled inflammation and oxidative stress may hold great therapeutic potential for treating this devastating condition.

Linoleic acid (LA) is a polyunsaturated essential fatty acid, characterized by the molecular formula CH3(CH2)4CH=CHCH2CH=CH(CH2)7COOH [6]. It is predominantly found in vegetable oils and nuts and is highly recommended for inclusion in the human diet. Previous research has demonstrated that LA exhibits diverse biological activities, including hypolipidemic effects, facilitation of cellular growth, promotion of weight loss, immune regulation, and others [7]. Clinical interventions have shown that dietary intake of LA reduces systemic inflammation and reduces the risk for cardiometabolic disease [8]. Prior studies have suggested the modulatory effects of LA on oxidative stress and inflammatory reaction [9]. A study of mast cells showed that LA effectively regulates the elevation of inflammatory mediators in activated mast cells [10]. However, whether supplementation with LA would modulate the development of ALI remains unclear.

Recently, mounting evidence indicates that abnormal mitochondrial functions are pivotal in ALI's development and progression [11]. Mitochondria are known as the powerhouses of cells. However, when their functions become impaired or dysregulated, it can lead to various pathological conditions. One crucial regulator responsible for upholding adequate mitochondrial function is peroxisome proliferator–activated receptor‐γ coactivator‐1α (PGC‐1α) [12]. PGC‐1α functions as a transcriptional coactivator that controls the expression of genes involved in the generation of new mitochondria. Nuclear respiratory factor 1 (NRF1) plays a crucial role in the regulation of mitochondrial function by overseeing the expression of genes responsible for encoding various components found within mitochondria, such as those involved in the electron transport chain and oxidative phosphorylation machinery [13]. These components are essential for efficient energy production by facilitating electron transfer and adenosine triphosphate (ATP) synthesis. In addition, the maintenance of mitochondrial integrity and functionality heavily relies on the crucial involvement of transcription factor A (TFAM) in mitochondria [14]. TFAM acts as a transcriptional activator for both nuclear‐encoded mitochondrial genes and its own gene. It ensures proper replication and maintenance of mitochondrial DNA (mtDNA). Together, these master regulators—PGC‐1α, NRF1, and TFAM—form an intricate network that orchestrates efficient mitochondrial production. Recent studies have shown that LA can prevent streptozotocin (STZ)‐induced rat RIN‐m5F cell death and enhance the mRNA and protein expression of PGC‐1α and TFAM in cells [15]. Similarly, a recent study has demonstrated that rrIL‐10 protects against PM2.5‐induced pulmonary injury by upregulating the expression of PGC‐1α [16]. Therefore, we investigated whether LA would up‐regulate the PGC‐1α/NRF1/TFAM signaling pathway expression, thereby reducing inflammation, inhibiting oxidative stress response.

## 2 Materials and Methods

### 2.1 Animals

The C57BL/6J male mice, aged 6–8 weeks and weighing approximately 18–22 g, were selected from the Experimental Animal Center of Chongqing Medical University. They had unrestricted access to food and water throughout the experiment to ensure their well‐being. The mice were housed in a climate‐controlled environment at a constant temperature of 25°C, with a 12‐h light/dark cycle mimicking natural day–night patterns. Before starting the actual experiment, we closely monitored the mice daily during an adaptation period until they had acclimated for over 1 week. Following meticulous protocols set by the Experimental Animal Center of Chongqing Medical University ensured both the welfare of our research subjects and reliable results from our experiments.

### 2.2 Model of ALI

Pre‐experiment: Mice were anesthetized with 50 mg/kg sodium pentobarbital and randomly divided into eight groups: control group, 20 mg/kg LA group, 50 mg/kg LA group, 100 mg/kg LA group, LPS group, LPS + 20 mg/kg LA group, LPS + 50 mg/kg LA group, and LPS + 100 mg/kg LA group. The control group was injected with 0.01 mL/g normal saline, and the LPS group was injected with 15 mg/kg LPS. LA was injected 1 h earlier than LPS. Three mice in each group were sacrificed after 18 h.

Formal experiment: The anesthetized mice were randomly divided into four groups: control group, LA (50 mg/kg) group, LPS group, and LA + LPS group. Twelve mice in each group were killed 18 h later. The other conditions were the same as the pre‐experiment.

### 2.3 Cell Culture and Treatment

The MLE‐12 cell line, derived from mouse lung epithelial cells, which was obtained from Procell Life Science & Technology. In order to maintain the cells in optimal conditions, they were cultivated in Dulbecco's modified Eagle's medium (DMEM), which was enhanced with 10% fatal bovine serum (FBS) and 1% penicillin–streptomycin solution. LA (≥ 99%, 18:2, n‐6, Sigma‐Aldrich, USA) was dissolved in ethanol and diluted in DMEM containing 2% bovine serum albumin (BSA, Beyotime, China) to final concentration as mentioned in previous reports. To prepare LPS (from *Escherichia coli* 055: B5, Sigma‐Aldrich, USA), it was mixed with sterile phosphate buffer saline (PBS). Similarly, SSR‐18292 (MedChemExpress, USA), a PGC‐1α inhibitor, was also dissolved in sterile PBS. The cells were cultured in six‐well plates for overnight and subsequently allocated into various experimental groups, including the control group (cultivated using serum‐free DMEM containing 2% BSA), LA group (treated with a concentration of 10 μM LA), LPS group (exposed to a concentration of 1000 ng/mL LPS), LA + LPS group (simultaneously treated with concentrations of 10 μM LA and 1000 ng/mL LPS), and LA + LPS + SR‐18292 group (administered concentrations of 10 μM LA, 1000 ng/mL LPS, and 20 μM SR‐18292). MLE‐12 cells in all experimental groups were incubated for a duration of 24 h.

### 2.4 Cell Viability

The impact of LA and LPS on cellular viability was evaluated by employing the Cell Counting Kit‐8 (CCK‐8, Dojindo, Japan) following established procedures. The MLE‐12 cells were carefully prepared by seeding them in well plates at an appropriate density to ensure optimal growth conditions. The MLE‐12 cells were cultured in a 96‐well plate with a seeding density of 5 × 10^3^ cells per well overnight and subsequently treated with various concentrations of LA (0, 2, 5, 10, 20, and 40 μM) or LPS (0, 100, 200, 500, and 1000 ng/mL) for a duration of 24 h. Following treatment, all wells were incubated with CCK‐8 reagent for a period of 1.5 h before measuring absorbance at a wavelength of 450 nm.

### 2.5 Behavioral Assessment Score

Mice of all groups were observed, in which the following mouse evaluation scores were used [17]. The scores were derived from the summation of four physical categories as follows: (A) coat, where 1 represents a smooth coat, 2 indicates a slightly ruffled one, and 3 signifies significant ruffling; (B) activity level, where 1 denotes normal activity levels, 2 suggests listlessness, 3 implies inactivity, and 4 means immobility; (C) respiratory effort (if breathing is normal, then it is scored as 1, but if it becomes laborious, then it is given a score of 2; if breathing becomes both laborious and irregular, then the score goes up to 3); and (D) posture (if an individual is moving or resting normally then they receive a score of 1, but if they are huddled up, then their score would be 2). Based on these four categories, total scores can range from 4 (indicating normalcy) to 12 (representing the least degree of normality).

### 2.6 Hematoxylin and Eosin (H&E)

A solution containing 4% formaldehyde was employed to fix the left lung tissue, ensuring that it remained in a stable and preserved state. Following this, sections of the tissue were subjected to staining with hematoxylin and subsequently restained with eosin. To further analyze these samples, histopathological examination was conducted using a high‐quality Leica Microsystems light microscope from Germany. After conducting a thorough analysis of the pathological features, which included congestion, edema, inflammation, and hemorrhage, we were able to categorize the histological changes on a scale ranging from 0 to 4 [15].

### 2.7 BALF

The mice were rendered painless by receiving an intraperitoneal injection of pentobarbital at a dosage of 50 mg/kg. The laryngeal tissue of the mouse was dissected; a needle was inserted into the exposed trachea and ligated with thread fixation. The lung of mouse was washed twice with 0.5 mL cold PBS. Finally, the samples were centrifuged at 3000 rpm/min for 10 min at 4°C. The supernatant was removed and stored in a refrigerator at −80°C.

### 2.8 Peritoneal Lavage Fluid (PLF)

After sacrificing the mice, 10 mL of pre‐cooled PBS was extracted using a syringe and injected into the abdominal cavity of each mouse. Gentle rubbing of the abdomen for 2 min facilitated proper distribution of PBS within the cavity. The mixture was allowed to stand for 5 min before withdrawing the fluid from the abdominal cavity. The lavage fluid samples were temporarily stored at 4°C.

### 2.9 Cell‐Free DNA (cfDNA)

Mouse cell‐free DNA (cfDNA) enzyme‐linked immunosorbent assay (ELISA) kit was purchased from Shanghai Win‐Win Biotechnology Co., Ltd., item number SY‐M01582. Experimental procedures were used according to the instructions.

### 2.10 Mitochondrial Membrane Potential (MMP)

Mitochondrial Membrane Potential (MMP) Detection Kit (TMRE) was purchased from Beyotime Biotechnology (C2001S). Experimental procedures were used according to the instructions.

### 2.11 ATP

ATP detection kit was purchased from Beyotime Biotechnology (S0026). Experimental procedures were used according to the instructions.

### 2.12 ELISA

In order to assess the concentrations of IL‐6, IL‐1β, and TNF‐α, we utilized ELISA kits from Neo Bioscience, a reputable manufacturer based in China. Following their instructions carefully, we were able to accurately quantify the concentrations of these important cytokines. Their absorbance was finally measured at 450 nm.

### 2.13 MPO Activity

The activity of MPO in lung tissue and BALF was then meticulously determined using a highly reliable MPO Detection Kit sourced from the esteemed Nanjing Jiancheng Bioengineering Institute in China. To determine the total protein concentration in lung samples, we used a protein assay kit (P0010, Beyotime Biotechnology, China) based on bicinchoninic acid (BCA).

### 2.14 SOD, MDA, and GSH Activity

The lung samples were homogenized in saline to ensure that the tissue was fully broken down and ready for analysis. To determine the activity present, we used assay kits provided by the Chinese Nanjing Jiancheng Bioengineering Institute. All trials were performed in accordance with the guidelines provided by the manufacturer. In addition, we quantified the total protein content present in each lung tissue sample using a BCA protein assay kit.

### 2.15 Western Blot

About 100 mg of lung tissue underwent lysis using 1 mL of RIPA lysis buffer (P0013B, Beyotime Biotechnology, China), followed by the addition of phosphatase inhibitor cocktail (P1081, Beyotime Biotechnology, China) and PMSF (ST506, Beyotime Biotechnology, China), at ratios equivalent to one part for every fifty parts and one part for every hundred parts, respectively. The MLE‐12 cells were harvested from six‐well plates, followed by treatment with 100 μL of RIPA lysate and thorough mixing through gentle blowing. The lysates were subsequently subjected to high speed of 14 000 × g for 5 min under cold conditions at 4°C. The BCA protein assay kit was employed according to the manufacturer's guidelines to determine the protein concentration in lung tissue and cells. After the protein extracts were subjected by SDS‐PAGE using gels of varying concentrations, including 7.5%, 12.5%, or 15%, the samples were moved to a high‐quality polyvinylidene fluoride (PVDF) membrane, which was sourced from Millipore in the United States. To prevent nonspecific binding of antibodies to the blots, PVDF was subjected to a thorough treatment with a solution containing 5% fat‐skimmed dry milk in Tris‐buffered saline (TBS) with 0.1% Tween 20. Finally, after blocking nonspecific sites on the blots, primary antibodies were incubated overnight at a low temperature of 4°C to detect target protein blots effectively. The primary antibodies utilized consisted of PGC‐1α (1:1000, BS‐1832R, Bioss, China), TFAM (1:1000, ab252432, Abcam, USA) and NRF1 (1:1000, ab175932, Abcam, USA). The band signals were subsequently captured using the FUSION SOLOS Imaging System (VILBER BIO IMAGING, France) and subjected to analysis using the ImageJ win64 software.

### 2.16 Measurement of mRNA Levels by Quantitative Reverse Transcription Polymerase Chain Reaction (RT‐PCR)

We utilized the TRIzol reagent, known for its exceptional efficiency, to extract total RNA from lung tissue and cells. The manufacturer's instructions were followed with great care during the process. The cDNA was synthesized utilizing the one‐step PCR reverse transcription kit (code.no. RR037A; Takara Bio, Inc.) and subsequently amplified by RT‐qPCR using TB Green Premix Ex Taq Kit (code.no. RR820A; Takara Bio, Inc.). The process was initiated by subjecting the sample to an initial denaturation step at a temperature of 95°C for a duration of 30 s, which helped to separate the double‐stranded DNA into single strands. Following this, a cycling protocol was employed that involved two key steps: denaturation at 95°C for a duration of only 5 s and annealing/extension at a lower temperature of 60°C that lasts for half a minute. This cycle was repeated a total of 40 times, allowing for the amplification of the target genes. To evaluate the relative expression of these genes, we utilized the highly effective and widely accepted method known as the “2^−ΔΔCq^” method. This approach allowed us to accurately compare gene expression levels across different samples by normalizing all data to our internal control β‐actin. These primers were synthesized commercially and are fully documented in Table 1, ensuring complete transparency and reproducibility of our methods.

**TABLE 1:** Primers used in this manuscript.

Gene	Forward primer (5′–3′)	Reverse primer (5′–3′)
IL‐6	AACCGCTATGAAGTTCCTCTCTG	TGGTATCCTCTGTGAAGTCTCCT
TNF‐α	CCTCACCCACACCGTCAG	CCTCACCCACACCGTCAG
β‐Actin	ACTGTCGAGTCGCGTCC	GTGACCCATTCCCACCATCA
PGC‐1α	AGTGAAGATGAAAGTGA	TTGAGAAAATAAGGATT
NRF1	ATGGGAAAGGTCGAATGGTATGT	CGTGCTCCTCCATGAAGATCTAC
TFAM	GGAGGCAAAGGATGATTCGG	CGTCCAACTTCAGCCATCTG

### 2.17 Statistical Analysis

The data analysis for this study was conducted using GraphPad Prism 8 to handle complex statistical analyses. To ensure accuracy and reliability of the results, we presented our findings as means ± standard deviation (SD). To compare multiple groups in our study, we employed one‐way analysis of variance (ANOVA) along with Tukey's post hoc test for multiple comparisons. It should be emphasized that statistical significance was considered when *p* < 0.05, indicating a low probability that any observed differences were due to chance alone. This threshold ensures that only truly meaningful results are reported and helps prevent false positives or misleading conclusions.

## 3 Results

### 3.1 50 mg/kg LA Exhibited Significant Protective Effects on Mice With ALI

**FIGURE 1:**
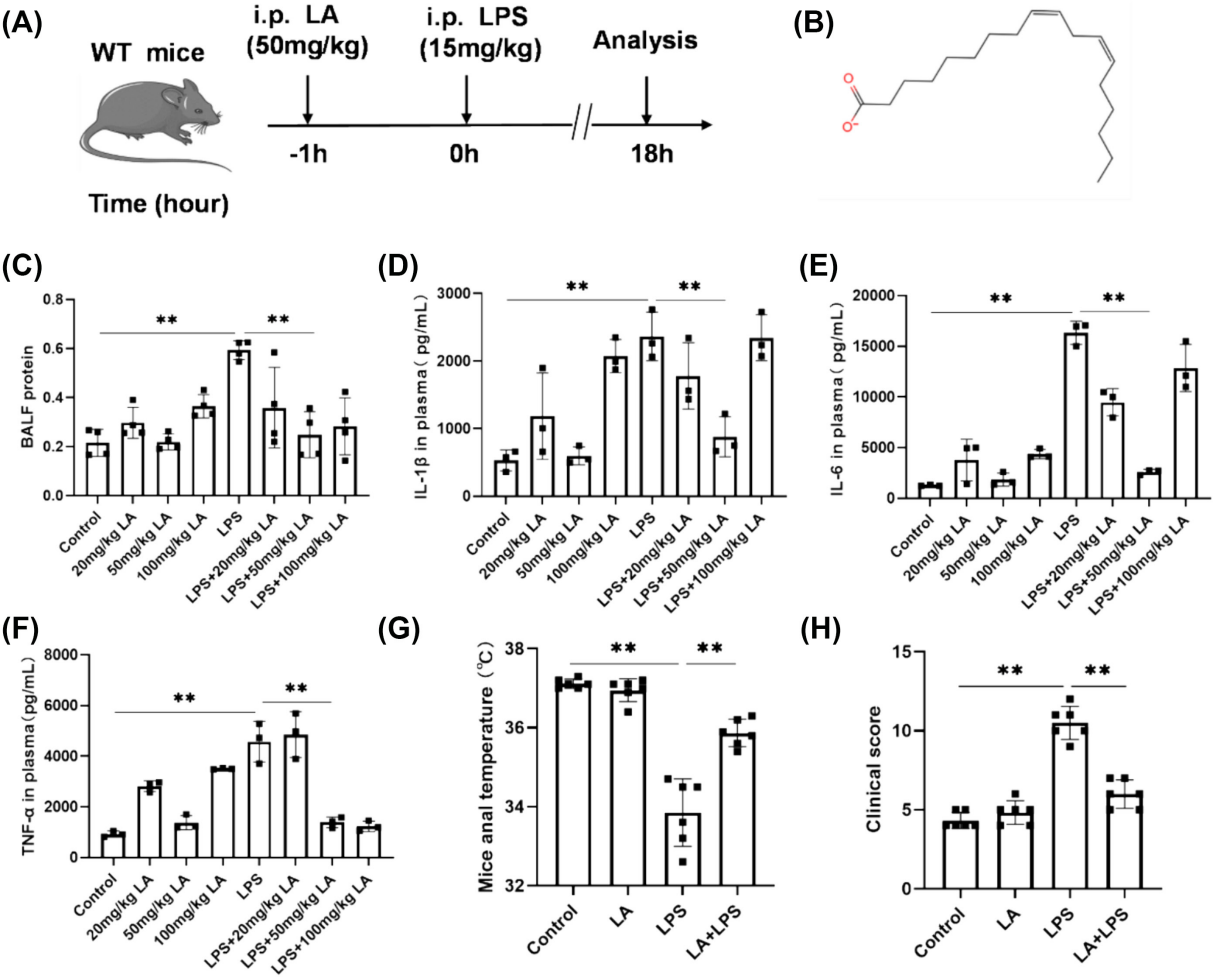
(A) The intraperitoneal injection and sampling time of mice model. (B) The chemical structure of LA. (C) BCA kit was utilized for quantifying the overall protein concentration in BALF. ELISA kits were employed to measure the quantification of inflammatory factors, including IL‐1β (D), IL‐6 (E), and TNF‐α (F) in plasma. (G) Among all groups, there was no significant difference in basal anal temperature. Anal temperature of mice in each group was measured after modeling. (H) The clinical score was evaluated based on four categories of physical appearance: coat, activity level, respiratory effort, and posture. We did each experiment three times and took the average with SD. **p < 0.01.

The mice were pretreated with three different concentrations of LA. As depicted in Figure 1C–F, pretreatment of ALI mice with a concentration of 50 mg/kg LA resulted in significant reductions in proinflammatory factor levels in plasma and BALF protein concentrations. However, the effects were not observed when using concentrations of 20 mg/kg and 100 mg/kg LA. Therefore, subsequent experiments employed LA at a concentration of 50 mg/kg.

### 3.2 LA Alleviated LPS‐Induced Systemic Abnormalities

Compared to the control group, we observed that LPS induced changes in systemic status, including disheveled fur, slowed movement, weakened response, and reduced respiration, which were evaluated with the clinical score. As expected, treatment with LA suppressed LPS‐induced upregulation of clinical score (Figure 1G), indicating improved systemic status of LPS‐exposed mice with LA treatment. Consistently, LPS‐induced hypothermia was also reversed by LA (Figure 1H).

### 3.3 LA Attenuated LPS‐Induced Neutrophil Release

**FIGURE 2:**
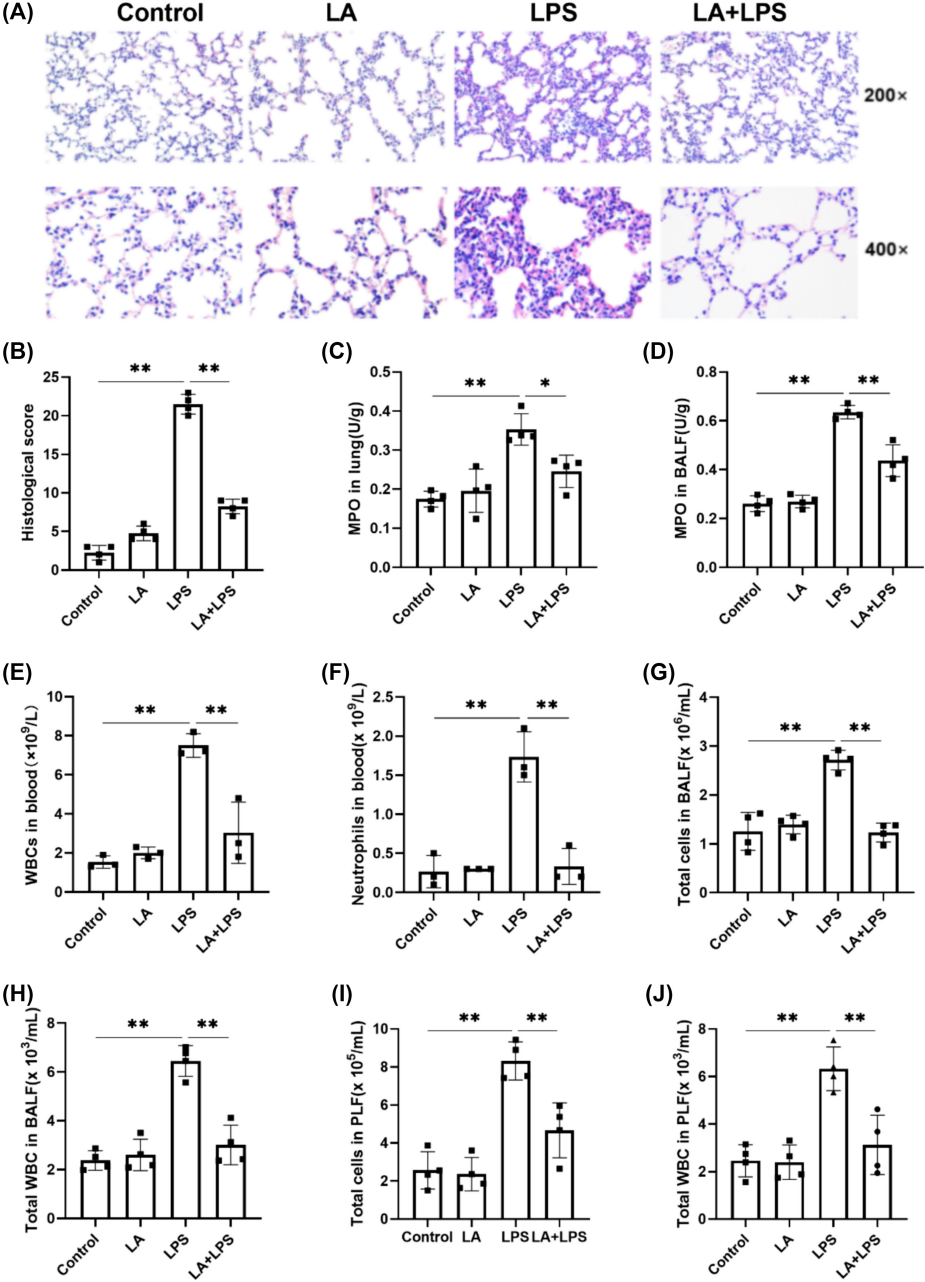
(A) These histological images depict left lung tissue stained with H&E at magnifications of 200× and 400×. (B) Histological scores were evaluated based: congestion, edema, inflammation, and hemorrhage. MPO Detection Kit was employed to assess MPO activity in both lung tissue (C) and BALF (D). The number of total cells (E), WBCs (F), and neutrophils (G) in blood were detected by automatic blood cell analyzer. The number of total cells (H,J) and WBCs (I,K) in blood and PLF were detected by automatic cell counter. We did each experiment three times and took the average with SD. *p < 0.05, **p < 0.01.

As indicated in Figure 2A,B, hematoxylin and eosin (H&E) staining results demonstrated that the control group displayed a typical structure, whereas those in the LPS group exhibited marked histopathological changes, including alveolar destruction, extensive inflammatory cell infiltration, and thickened alveolar septum. However, LA pretreatment significantly reduced these pathological features. As depicted in Figure 2C,D, LA pretreatment resulted in a decrease of MPO activity induced by LPS in both lung tissues and BALF. Similarly, as depicted in Figure 2E–J, pretreatment with LA attenuated the LPS‐induced elevation in WBC and neutrophil count in peripheral blood, as well as mitigating LPS‐induced increases in total cell and leukocyte counts in BALF and PLF. Collectively, these results supported that LA could reduce neutrophil release.

### 3.4 LA Suppressed LPS‐Induced Inflammatory Cytokine Release

**FIGURE 3:**
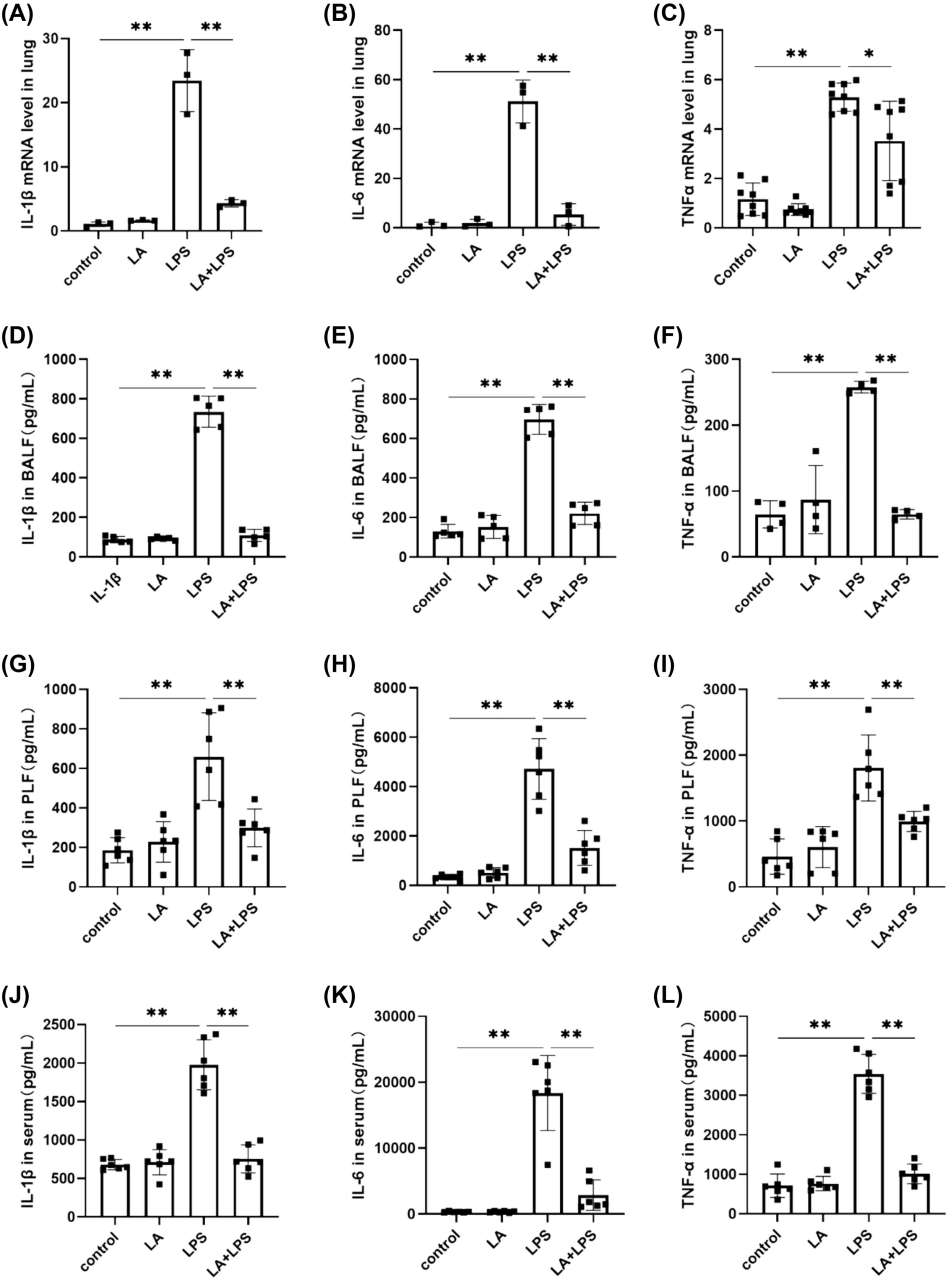
PCR was conducted to assess the expression levels of IL‐1β (A), IL‐6 (B), and TNF‐α (C) mRNA in lung tissue. ELISA kits were employed to measure the quantification of inflammatory factors, including IL‐1β (D,G,J), IL‐6 (E, H,K), and TNF‐α (F,I,L) in BALF, PLF, and serum. We did each experiment three times and took the average with SD. *p < 0.05, **p < 0.01.

As shown in Figure 3A–C, the results demonstrated a significant increase in the mRNA expressions of IL‐6, IL‐1β, and TNF‐α in lung of the LPS group, although LA pretreatment resulted in a notable reduction. Similarly, according to Figure 3D–L, the elevation of IL‐6, IL‐1β, and TNF‐α induced by LPS was significantly ameliorated after LA pretreatment in BALF, PLF, and serum. These findings indicated that LA could relieve lung and systemic inflammation.

### 3.5 LA Alleviated LPS‐Induced Remote Damage and Lung Injury

**FIGURE 4:**
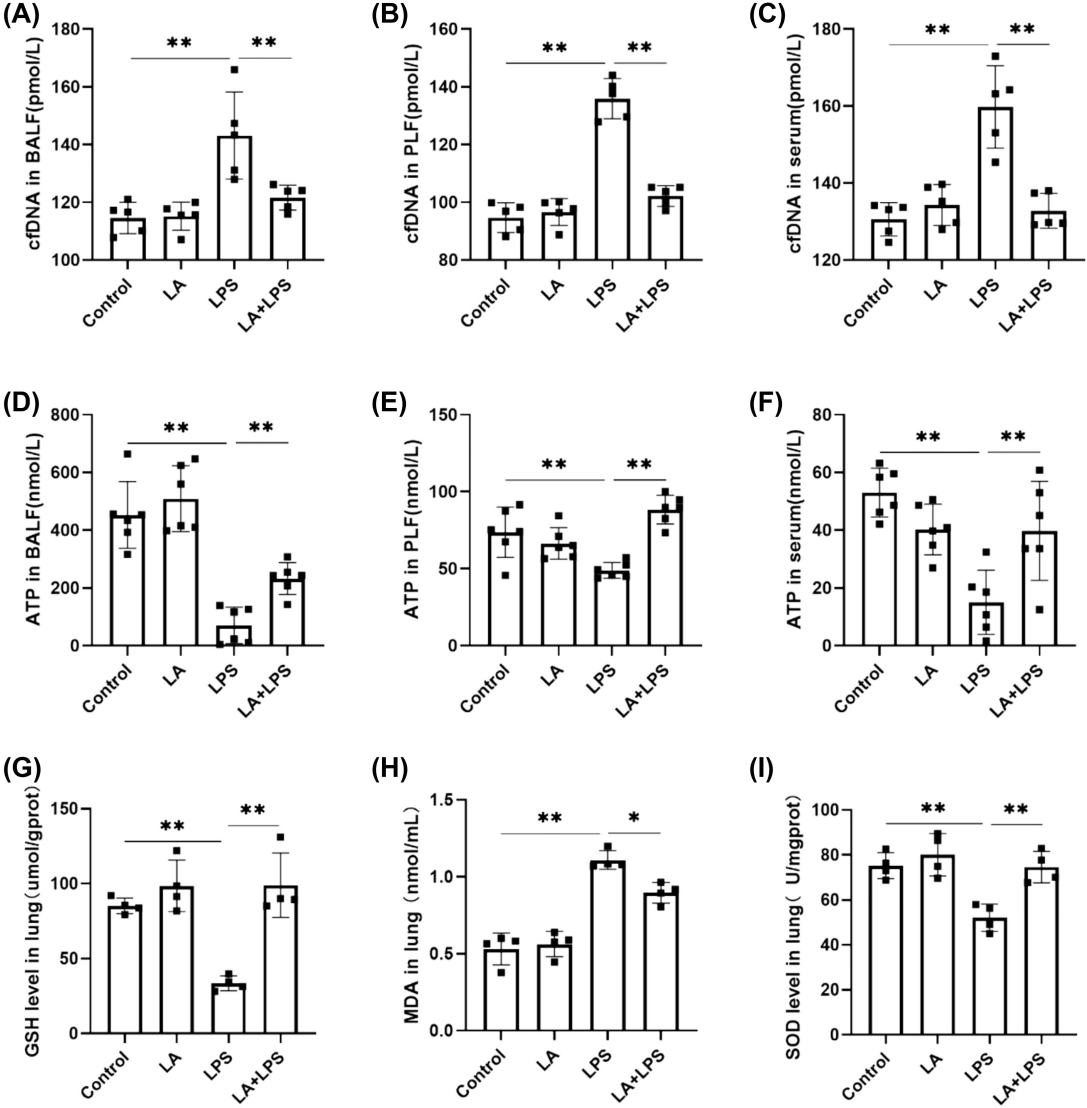
The concentration of cfDNA in BALF (A), PLF (B), and serum (C) was measured with a mouse cfDNA ELISA kit. ATP detection kits were used to detect the concentration of ATP in the BALF (D), PLF (E), and serum (F). The levels of GSH (G), MDA (H), and SOD (I) in mice lungs were tested by commercialized kits. We did each experiment three times and took the average with SD. *p < 0.05, **p < 0.01.

LPS led to the liberation of cfDNA from BALF, PLF, and serum, as well as a reduction in ATP production. However, these pathological processes were reversed by pretreatment with LA (Figure 4A–F).

### 3.6 LA Inhibited LPS‐Induced Oxidative Stress

In Figure 4G–I, the concentration of MDA, a biomarker indicative of lipid peroxidation, exhibited a significant increase in lung tissue in the LPS group compared to the control group. Conversely, both SOD and GSH, possessing antioxidative properties, demonstrated a notable decrease. However, SOD and GSH levels showed a remarkable increase after LA treatment, along with MDA levels showing a significant decrease.

### 3.7 LA Promoted PGC‐1α/NRF1/TFAM Expression in ALI Mice

**FIGURE 5:**
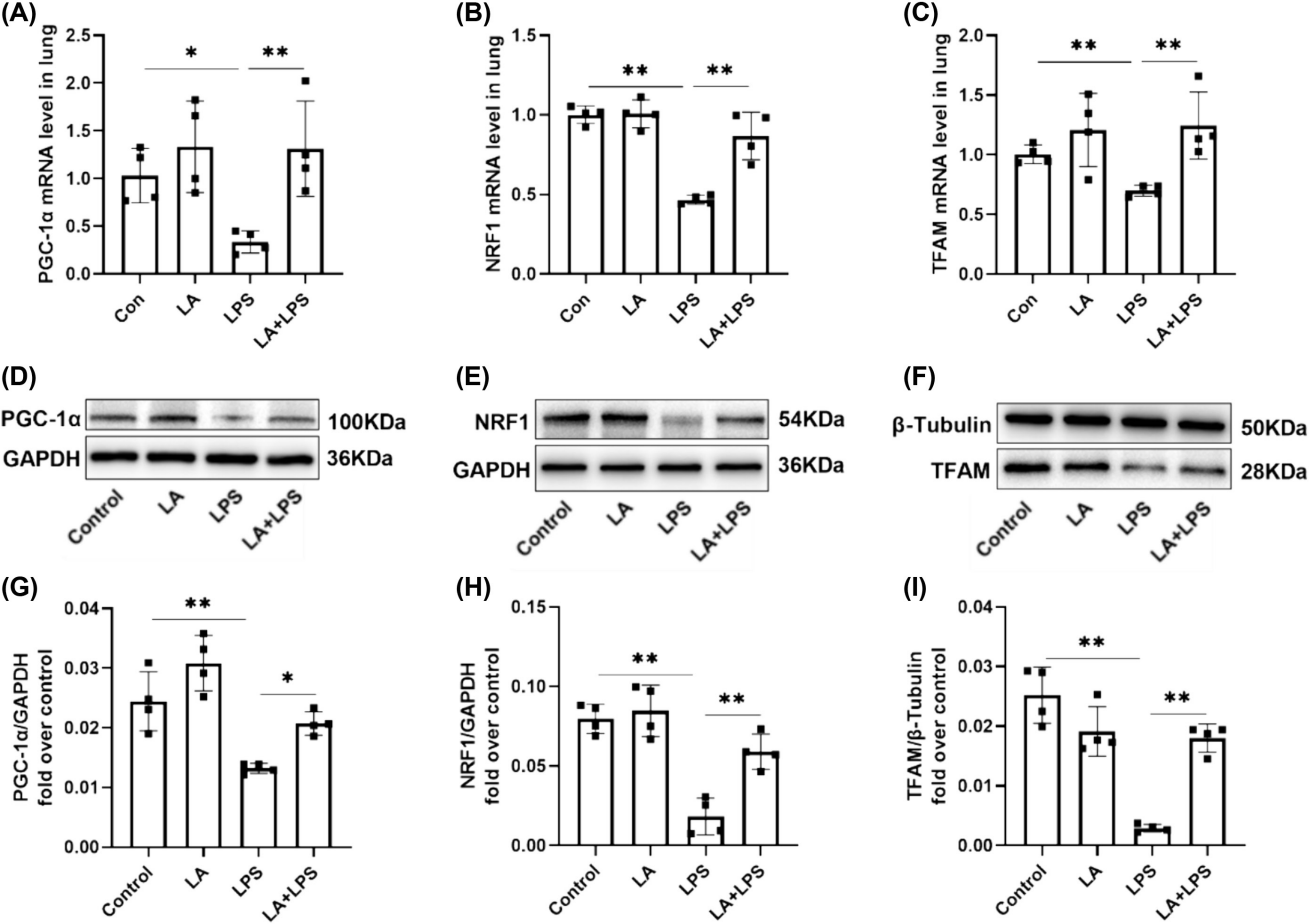
PCR was employed to evaluate the mRNA levels of PGC‐1α (A), NRF1 (B) and TFAM (C) essential for mitochondrial biogenesis in mouse lung tissue. Lung homogenates were prepared, followed by western blotting using specific antibodies against PGC‐1α (D, G), NRF1 (E, H) and TFAM (F, I). The values below each lane were normalized relative to the intensity of the GAPDH or β‐Tubulin band. We did each experiment three times and took the average with SD. *p < 0.05, **p < 0.01.

It has been well documented that activating the PGC1‐α/NRF1/TFAM signaling axis enhances mitochondrial biogenesis. The results in Figure 5A–C show a significant decrease in mRNA expression levels of PGC‐1α, NRF1, and TFAM in the LPS group. However, pretreatment with LA markedly increased the levels of these mRNAs. Consistently, in Figure 5D–I, WB results demonstrated a significant decrease in PGC‐1α, NRF1, and TFAM proteins in the LPS group. However, LA pretreatment led to a significant increase in these protein levels.

### 3.8 LA Reduced Inflammatory Cytokine Release in MLE‐12 Cell

**FIGURE 6:**
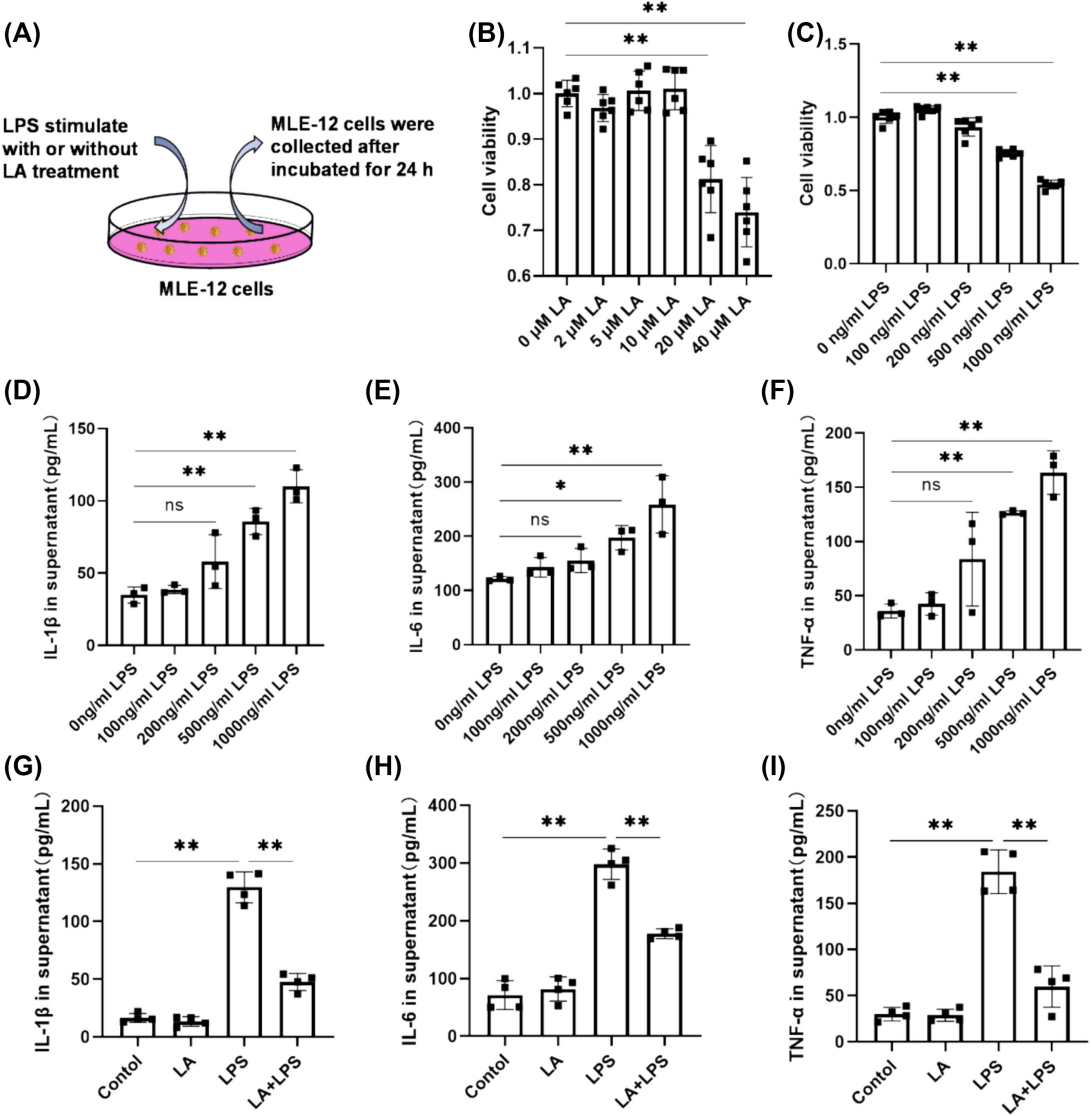
(A) Cell model. (B) The survival rate of MLE‐12 cells following treatment with varying concentrations of LA. (C) The survival rate of MLE‐12 cells exposed to varying levels of LPS. The levels of IL‐6 (D), IL‐1β (E), and TNF‐α (F) in the cell supernatant were measured using an ELISA kit following stimulation with varying concentrations of LPS. The concentrations of IL‐6 (G), IL‐1β (H), and TNF‐α (I) in the supernatant from each experimental group of cells were detected by ELISA. We did each experiment three times and took the average with SD. *p < 0.05, **p < 0.01.

An in vitro ALI model was established by stimulating MLE‐12 cells with LPS. CCK‐8 assay results showed the content of 10 μM LA had minimal impact on cell viability, as shown in Figure 5B. Therefore, we chose this concentration (10 μM LA) for subsequent investigations. The impact of LPS stimulation at various concentrations on MLE‐12 cells was assessed. As depicted in Figure 6C–F, upon stimulation with 1000 ng/mL LPS, MLE‐12 cell viability significantly decreased accompanied by the most pronounced inflammatory response. Therefore, this concentration (1000 ng/mL) was selected for subsequent experiments. ELISA results demonstrated that LA could mitigate the release of proinflammatory cytokines induced by LPS, including IL‐1β, IL‐6, and TNF‐α (Figure 6G–I).

### 3.9 LA Alleviated LPS‐Induced Mitochondrial Dysfunction

**FIGURE 7:**
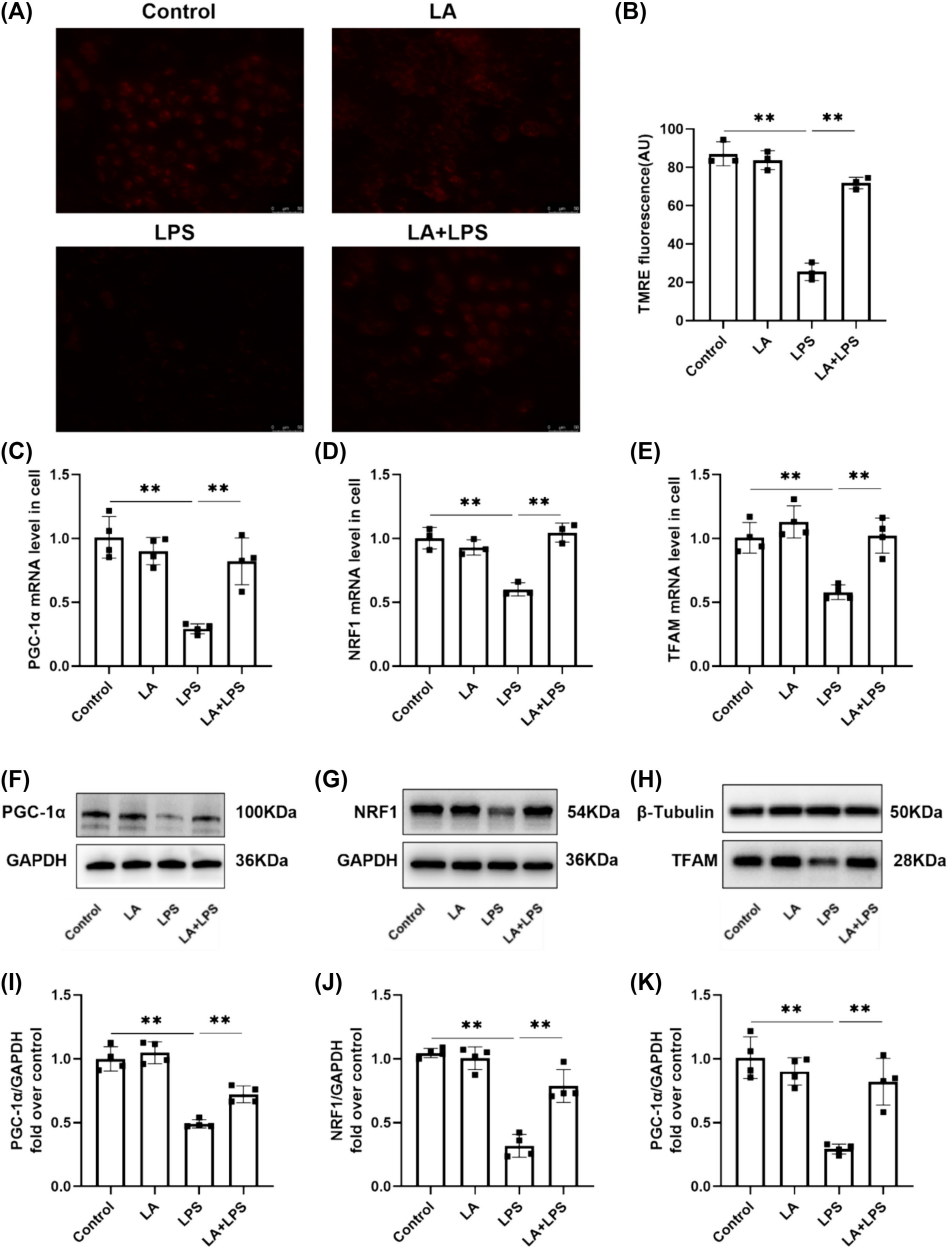
(A) MMP of MLE‐12 cells was detected by TMRE, and the above four groups of cells were observed by fluorescence microscope. Scale bar, 10 μm. (B) The fluorescence intensity was analyzed by ImageJ. PCR for the assessment of the mRNA levels of PGC‐1α (C), NRF1 (D), and TFAM (E) necessary for mitochondrial biogenesis in cells. Homogenates of cells were prepared and western blotting was performed using PGC‐1α (F,I), NRF1 (G,J), and TFAM (H,K)‐specific antibodies. The values below each lane are normalized relative to the intensity of the GAPDH or β‐tubulin band. We did each experiment three times and took the average with SD. **p < 0.01.

Fluorescence microscopy showed that the red fluorescence became weaker in the LPS group, indicating a decrease in mitochondrial membrane potential, whereas the red fluorescence became stronger after LA treatment (Figure 7A,B). The results of the PCR analysis demonstrated a decrease in the mRNA expression of PGC‐1α, NRF1, and TFAM upon LPS stimulation. However, LA treatment significantly improved mitochondrial dysfunction in MLE‐12 cells (Figure 7C–E). Western blot analysis consistently revealed that LA treatment mitigated LPS‐induced reduction in protein expression of PGC‐1α, NRF1, and TFAM (Figure 7F–K). In conclusion, LA effectively alleviated LPS‐induced mitochondrial dysfunction in MLE‐12 cells.

### 3.10 Inhibition of PGC‐1α Partially Blocked the Effect of Linoleic Acid on ALI Treatment in Cells

**FIGURE 8:**
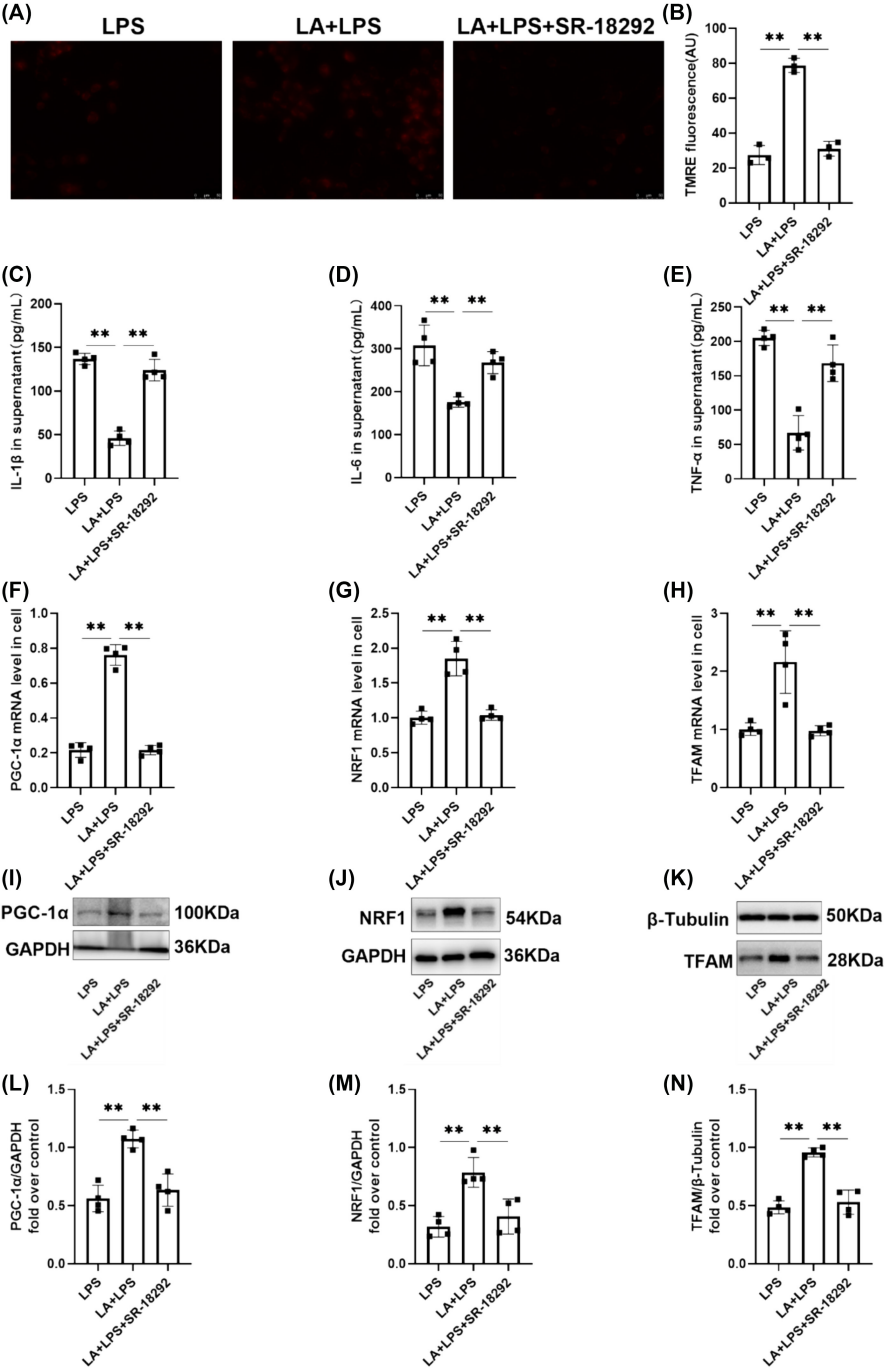
(A) MMP of MLE‐12 cells was detected by TMRE, and the above three groups of cells were observed by fluorescence microscope. Scale bar, 10 μm. (B) The fluorescence intensity was analyzed by ImageJ. The concentrations of IL‐1β (C), IL‐6 (D), and TNF‐α (E) in cell supernatant were measured by ELISA. RT‐qPCR for the assessment of the mRNA levels of PGC‐1α (F), NRF1 (G), and TFAM (H) necessary for mitochondrial biogenesis in cells. Homogenates of cells were prepared and western blotting was performed using PGC‐1α (I,L), NRF1 (J,M), and TFAM (K,N)‐specific antibodies. The values below each lane are normalized relative to the intensity of the GAPDH or β‐tubulin band. We did each experiment three times and took the average with SD. **p < 0.01.

The treatment of the cells with the PGC‐1α inhibitor SR‐18292 attenuated the stimulatory effect of LA on MMP (Figure 8A,B) and the amelioration of inflammatory cytokine release (Figure 8C–E). In comparison to the LPS group, both mRNA and protein levels of PGC‐1α, NRF1, and TFAM were significantly elevated in the LA + LPS group. However, following SR‐18292 treatment, there was no significant difference observed between the LPS group and LA + LPS + SR‐18292 group (Figure 8D–I). Collectively, these findings suggest that PGC‐1α inhibitors partially impede the restorative impact of LA on mitochondrial biogenesis.

## 4 Discussion

ALI is a condition that poses significant risks to health and has been linked to increased rates of illness and death. LPS‐induced pulmonary injury remains a significant cause of death, lacking satisfactory treatment options and cure. Therefore, the development of novel drugs for ALI treatment is imperative. LA, a polyunsaturated fatty acid abundantly present in natural nuts and vegetables, exhibits anti‐inflammatory and antioxidant properties. To our knowledge, no previous studies have investigated the effects of LA in relation to LPS‐induced lung injury. Alveolar epithelial cells have a crucial function in the preservation of tissue homeostasis, making them widely utilized as a model for investigating lung diseases and cellular responses to diverse stimuli. For the purpose of examining the potential impact of LA on ALI, we developed in vitro and in vivo models, which included an LPS‐induced mouse ALI model and an LPS‐induced MLE‐12 cell model. The findings indicate that the participation of PGC‐1α/NRF1/TFAM is responsible for the beneficial impact of LA in mitigating ALI induced by LPS.

Uncontrolled inflammation has been regarded to be responsible for the progress of ALI [18]. LA is an essential fatty acid that is widely present in people's diets [19]. For example, both the American Heart Association and the Dietary Guidelines for Americans recommend intake of LA. In our study, LPS caused severe lung injury in mice. Inflammatory markers such as IL‐6, IL‐1β, and TNF‐α in lung tissue, plasma, serum, BALF, and PLF of mice were increased. The staining of lung tissue showed inflammatory cell aggregation, increased hemorrhage, and pulmonary edema. Increased MPO activity in lung tissue and BALF. The number of white blood cells increased in peripheral blood, BALF, and PLF. The concentration of cfDNA was increased and ATP content was decreased in BALF, PLF, and serum. These results all indicate an exacerbation of lung injury and systemic inflammation. The administration of LA can effectively reduce LPS‐induced ALI lung injury and improve the above adverse reactions. Similarly, previous studies have suggested that LA might exert beneficial roles in several inflammation‐based disorders including insulin resistance [20], coronary heart disease [21], and cancer [22]. Therefore, these findings underlined anti‐inflammatory properties of LA.

The crucial involvement of oxidative stress in the pathological advancement of ALI has been substantiated [23]. In our study, oxidative factors including MDA, GSH, and SOD in lung tissues were tested to determine the antioxidant activities of LA. The results indicate that LPS‐induced oxidation stress was significantly suppressed by treatment with LA. This is consistent with previous studies conducted on a large yellow croaker model, where LA intake was found to enhance liver antioxidant performance [24]. Based on our study results, it can be suggested that LA might promote LPS‐induced ALI via limitation of oxidation stress.

Mitochondria is an important organelle of the body. In addition to producing direct energy ATP for cell life activities, regulating cell apoptosis, and maintaining the balance of electrolyte homeostasis, mitochondria are also responsible for the generation of oxygen free radicals in cells, regulating cell redox reaction and signal transduction. The mitochondria serve as the primary source of ROS generation and are predominantly susceptible to ROS assault. PGC‐1α is an important molecule that regulates mitochondrial biosynthesis. LPS exposure may result in an excessive rise in ROS levels, thereby disrupting the expression of PGC‐1α, impeding the antioxidative capabilities of mitochondria, and causing impairment to mitochondrial function. Reducing ROS can promote the expression of PGC‐1α and induce mitochondrial biosynthesis to play a role in mitochondrial protection [25]. The excessive rise in ROS levels leads to an imbalance between mitochondrial fusion and fission, which significantly contributes to the advancement of multiple organ failure syndrome linked with ALI [26, 27]. The process of mitochondrial production is a highly intricate physiological phenomenon that is primarily governed by nuclear and mitochondrial factors, including the master regulators PGC‐1, NRF1 [28], and the essential transcriptional activator TFAM [29]. It has been reported that in activated B cells, PGC‐1α inactivates nuclear factor kappa light chain enhancer (NFκB), reduces inflammatory responses, and participates in mitochondrial biogenesis by controlling NRF1 and TFAM [30]. Therefore, the inhibition of mitochondrial biosynthesis is intricately linked to the escalation of inflammation and oxidative stress in ALI. Our study has revealed some fascinating insights into the effects of LPS and LA on mRNA expression in relation to mitochondrial biogenesis. Our PCR findings indicate that the expression of PGC‐1α, NRF1, and TFAM—crucial regulators involved in mitochondrial biosynthesis—is significantly suppressed by LPS. However, we found that treatment with LA effectively reversed this effect. To further validate our findings, we conducted western blot analysis, which confirmed that activation of the PGC‐1α/NRF1/TFAM axis promotes mitochondrial DNA replication and transcription.

The alveolar epithelium functions as a protective shield, separating the organism from the surroundings and playing a crucial role in safeguarding against respiratory pathogens. Maintaining homeostasis in alveolar epithelial cells is crucial for pulmonary respiration and lung function. Evidence increasingly supports that ALI involves damage to these cells, emphasizing the importance of timely repair for treating ALI. Therefore, the MLE‐12 cell line is a valuable tool for studying lung diseases and cellular responses to different stimuli. Derived from mouse distal respiratory epithelial cells, this cell line closely resembles human alveolar type II cells and exhibits similar characteristics. Our findings demonstrated that the administration of LA reduced inflammation induced by LPS and improved mitochondrial dysfunction caused by LPS in cells. Interestingly, we found that the protective effect of LA on cells was abolished after administration of PGC‐1α inhibitor. Its inhibition suggests that the beneficial effects of LA on cell protection may be mediated through PGC‐1α signaling pathways.

In this study, we observed that LA could indeed exert a lung protective effect, and the possible protective mechanism of LA was confirmed by detecting the changes in the levels of proteins related to mitochondrial dysfunction and cytokines related to inflammation. It provides some new ideas and methods for the treatment of ALI in the future. At the same time, we also used PGC‐1α inhibitor in cell experiments to observe that the protective effect was antagonized. However, it is difficult to identify the specific targets of action of specific LA. LA is mainly absorbed through the intestine. In this study, we used the method of intraperitoneal injection of LA to treat mice, and the concentration of LA in plasma or alveolar epithelial cells was not measured, so the effectiveness of the method of intraperitoneal injection of LA cannot be determined.

Collectively, these findings suggest that LA can effectively alleviate ALI by activating the PGC‐1α/NRF1/TFAM pathway, promoting mitochondrial biogenesis, reducing oxidative stress levels, suppressing proinflammatory factors, and conferring protection against ALI in animal and cell models.

## Author Contributions

Conceived and designed the experiments: Gang Liu and Li Zhang. Performed the experiments: Jie Liu and Yu Jiang. Analyzed the data: Jie Liu, Yu Jiang, Qiuhong Zhang, and Yin Qin. Contributed reagents/materials/analysis tools and provided constructive discussion: Jie Liu, Yu Jiang, Kexin Li, Xiaoliang Wang, Tingting Zhang, and Xi Yang. Wrote and revised the manuscript: Jie Liu, Yu Jiang Gang Liu, and Li Zhang. All authors have read and approved the manuscript.

## Ethics Statement

The animal study protocol was approved by the Ethics Committee of University‐Town Hospital of Chongqing Medical University, Chongqing, China (protocol code LL‐202238 and approval date 21 December 2021), and consent to participate and publish the article was obtained.

## Conflicts of Interest

The authors declare no conflicts of interest.

## Data Availability

The data that support the findings of this study are available at corresponding author upon reasonable request.

## References

[1] R. G. Spragg , G. R. Bernard , W. Checkley , et al., “Beyond Mortality: Future Clinical Research in Acute Lung Injury,” American Journal of Respiratory and Critical Care Medicine 181, no. 10 (2010): 1121–1127. 20224063 10.1164/rccm.201001-0024WS PMC2874454

[2] C. M. Shaver and J. A. Bastarache , “Clinical and Biological Heterogeneity in Acute Respiratory Distress Syndrome: Direct Versus Indirect Lung Injury,” Clinics in Chest Medicine 35, no. 4 (2014): 639–653. 25453415 10.1016/j.ccm.2014.08.004 PMC4254695

[3] R. B. Goodman , J. Pugin , J. S. Lee , and M. A. Matthay , “Cytokine‐Mediated Inflammation in Acute Lung Injury,” Cytokine & Growth Factor Reviews 14, no. 6 (2003): 523–535. 14563354 10.1016/s1359-6101(03)00059-5

[4] V. Mirakaj , C. A. Thix , S. Laucher , et al., “Netrin‐1 Dampens Pulmonary Inflammation During Acute Lung Injury,” American Journal of Respiratory and Critical Care Medicine 181, no. 8 (2010): 815–824. 20075388 10.1164/rccm.200905-0717OC

[5] K. Vaporidi , C. Tsatsanis , D. Georgopoulos , and P. N. Tsichlis , “Effects of Hypoxia and Hypercapnia on Surfactant Protein Expression Proliferation and Apoptosis in A549 Alveolar Epithelial Cells,” Life Sciences 78, no. 3 (2005): 284–293. 16125734 10.1016/j.lfs.2005.04.070

[6] G. L. Russo , “Dietary n‐6 and n‐3 Polyunsaturated Fatty Acids: From Biochemistry to Clinical Implications in Cardiovascular Prevention,” Biochemical Pharmacology 77, no. 6 (2009): 937–946. 19022225 10.1016/j.bcp.2008.10.020

[7] L. Ferrucci , A. Cherubini , S. Bandinelli , et al., “Relationship of Plasma Polyunsaturated Fatty Acids to Circulating Inflammatory Markers,” Journal of Clinical Endocrinology and Metabolism 91, no. 2 (2006): 439–446. 16234304 10.1210/jc.2005-1303

[8] M. A. Belury , “Linoleic Acid, an Omega‐6 Fatty Acid That Reduces Risk for Cardiometabolic Diseases: Premise, Promise and Practical Implications,” Current Opinion in Clinical Nutrition and Metabolic Care 26, no. 3 (2023): 288–292. 37017716 10.1097/MCO.0000000000000919

[9] J. K. Innes and P. C. Calder , “Omega‐6 Fatty Acids and Inflammation,” Prostaglandins, Leukotrienes and Essential Fatty Acids 132 (2018): 41–48. 29610056 10.1016/j.plefa.2018.03.004

[10] H. Y. Kim , S. H. Rho , J. H. Lim , H. J. Park , and H. J. Jeong , “Protective Effect of Linoleic Acid Against Inflammatory Reactions by Mast Cell via Caspase‐1 Cascade Pathways,” Journal of Food Biochemistry 43, no. 8 (2019): e12932. 31368553 10.1111/jfbc.12932

[11] W. Liu , Y. Li , L. Bo , C. Li , and F. Jin , “Positive Regulation of TFEB and Mitophagy by PGC‐1α to Alleviate LPS‐Induced Acute Lung Injury in Rats,” Biochemical and Biophysical Research Communications 577 (2021): 1–5. 34482051 10.1016/j.bbrc.2021.08.064

[12] C. Handschin and B. M. Spiegelman , “The Role of Exercise and PGC1α in Inflammation and Chronic Disease,” Nature 454, no. 7203 (2008): 463–469. 18650917 10.1038/nature07206 PMC2587487

[13] Y. Li , W. W. Luo , X. Cheng , et al., “Curcumin Attenuates Isoniazid‐Induced Hepatotoxicity by Upregulating the SIRT1/PGC‐1α/NRF1 Pathway,” Journal of Applied Toxicology 42, no. 7 (2022): 1192–1204. 35032049 10.1002/jat.4288

[14] J. Xiao , C. Wu , Y. He , et al., “Rice Bran Phenolic Extract Confers Protective Effects Against Alcoholic Liver Disease in Mice by Alleviating Mitochondrial Dysfunction via the PGC‐1α‐TFAM Pathway Mediated by microRNA‐494‐3p,” Journal of Agricultural and Food Chemistry 68, no. 44 (2020): 12284–12294. 33094608 10.1021/acs.jafc.0c04539

[15] R. Ogata , S. Mori , S. Kishi , et al., “Linoleic Acid Upregulates Microrna‐494 to Induce Quiescence in Colorectal Cancer,” International Journal of Molecular Sciences 23, no. 1 (2021): 225. 35008652 10.3390/ijms23010225 PMC8745195

[16] N. Zhang , P. Li , H. Lin , et al., “IL‐10 Ameliorates PM2.5‐Induced Lung Injury by Activating the AMPK/SIRT1/PGC‐1alpha Pathway,” Environmental Toxicology and Pharmacology 86 (2021): 103659. 33862202 10.1016/j.etap.2021.103659

[17] J. M. Konter , J. L. Parker , E. Baez , et al., “Adiponectin Attenuates Lipopolysaccharide‐Induced Acute Lung Injury Through Suppression of Endothelial Cell Activation,” Journal of Immunology 188, no. 2 (2012): 854–863. 10.4049/jimmunol.1100426 PMC3253176 22156343

[18] M. A. Matthay , R. L. Zemans , G. A. Zimmerman , et al., “Acute Respiratory Distress Syndrome,” Nature Reviews. Disease Primers 5, no. 1 (2019): 18. 10.1038/s41572-019-0069-0 PMC6709677 30872586

[19] Y. Wu , G. Sun , X. Zhou , et al., “Pregnancy Dietary Cholesterol Intake, Major Dietary Cholesterol Sources, and the Risk of Gestational Diabetes Mellitus: A Prospective Cohort Study,” Clinical Nutrition 39, no. 5 (2020): 1525–1534. 31296343 10.1016/j.clnu.2019.06.016

[20] F. Imamura , R. Micha , J. H. Y. Wu , et al., “Effects of Saturated Fat, Polyunsaturated Fat, Monounsaturated Fat, and Carbohydrate on Glucose‐Insulin Homeostasis: A Systematic Review and Meta‐Analysis of Randomised Controlled Feeding Trials,” PLoS Medicine 13, no. 7 (2016): e1002087. 27434027 10.1371/journal.pmed.1002087 PMC4951141

[21] W. C. Willett , “The Role of Dietary n‐6 Fatty Acids in the Prevention of Cardiovascular Disease,” Journal of Cardiovascular Medicine (Hagerstown, Md.) 8, no. Suppl 1 (2007): S42–S45. 17876199 10.2459/01.JCM.0000289275.72556.13

[22] J. Li , M. Guasch‐Ferré , Y. Li , and F. B. Hu , “Dietary Intake and Biomarkers of Linoleic Acid and Mortality: Systematic Review and Meta‐Analysis of Prospective Cohort Studies,” American Journal of Clinical Nutrition 112, no. 1 (2020): 150–167. 32020162 10.1093/ajcn/nqz349 PMC7326588

[23] B. N. Whitley , E. A. Engelhart , and S. Hoppins , “Mitochondrial Dynamics and Their Potential as a Therapeutic Target,” Mitochondrion 49 (2019): 269–283. 31228566 10.1016/j.mito.2019.06.002 PMC6885535

[24] B. Yang , Y. Zhou , M. Wu , X. Li , K. Mai , and Q. Ai , “Omega‐6 Polyunsaturated Fatty Acids (Linoleic Acid) Activate Both Autophagy and Antioxidation in a Synergistic Feedback Loop via TOR‐Dependent and TOR‐Independent Signaling Pathways,” Cell Death & Disease 11, no. 7 (2020): 607. 32732901 10.1038/s41419-020-02750-0 PMC7393504

[25] T. L. Marin , B. Gongol , F. Zhang , et al., “AMPK Promotes Mitochondrial Biogenesis and Function by Phosphorylating the Epigenetic Factors DNMT1, RBBP7, and HAT1,” Science Signaling 10, no. 464 (2017): eaaf7478. 28143904 10.1126/scisignal.aaf7478 PMC5830108

[26] H. Wang , X. Sun , Q. Lu , et al., “The Mitochondrial Redistribution of eNOS Is Involved in Lipopolysaccharide Induced Inflammasome Activation During Acute Lung Injury,” Redox Biology 41 (2021): 101878. 33578126 10.1016/j.redox.2021.101878 PMC7879038

[27] W. Li , M. Li , K. Chen , et al., “Oxaloacetate Acid Ameliorates Paraquat‐Induced Acute Lung Injury by Alleviating Oxidative Stress and Mitochondrial Dysfunction,” Frontiers in Pharmacology 13 (2022): 1029775. 36313362 10.3389/fphar.2022.1029775 PMC9606601

[28] S. Jäger , C. Handschin , J. St.‐Pierre , and B. M. Spiegelman , “AMP‐Activated Protein Kinase (AMPK) Action in Skeletal Muscle via Direct Phosphorylation of PGC‐1alpha,” Proceedings of the National Academy of Sciences of the United States of America 104, no. 29 (2007): 12017–12022. 17609368 10.1073/pnas.0705070104 PMC1924552

[29] E. Nisoli , E. Clementi , C. Paolucci , et al., “Mitochondrial Biogenesis in Mammals: The Role of Endogenous Nitric Oxide,” Science 299, no. 5608 (2003): 896–899. 12574632 10.1126/science.1079368

[30] N. Ivanovski , H. Wang , H. Tran , et al., “L‐Citrulline Attenuates Lipopolysaccharide‐Induced Inflammatory Lung Injury in Neonatal Rats,” Pediatric Research 94, no. 5 (2023): 1684–1695. 37349511 10.1038/s41390-023-02684-1

